# Association of puberty timing with type 2 diabetes: A systematic review and meta-analysis

**DOI:** 10.1371/journal.pmed.1003017

**Published:** 2020-01-06

**Authors:** Tuck Seng Cheng, Felix R. Day, Rajalakshmi Lakshman, Ken K. Ong

**Affiliations:** 1 MRC Epidemiology Unit, Institute of Metabolic Science, University of Cambridge, Cambridge, United Kingdom; 2 Department of Paediatrics, University of Cambridge, Cambridge, United Kingdom; Chinese University of Hong Kong, CHINA

## Abstract

**Background:**

Emerging studies have investigated the association between puberty timing, particularly age at menarche (AAM), and type 2 diabetes. However, whether this association is independent of adiposity is unclear. We aimed to systematically review published evidence on the association between puberty timing and type 2 diabetes (T2D) or impaired glucose tolerance (IGT), with and without adjustment for adiposity, and to estimate the potential contribution of puberty timing to the burden of T2D in the United Kingdom (UK).

**Methods and findings:**

We searched PubMed, Medline, and Embase databases for publications until February 2019 on the timing of any secondary sexual characteristic in boys or girls in relation to T2D/IGT. Inverse-variance-weighted random-effects meta-analysis was used to pool reported estimates, and meta-regression was used to explore sources of heterogeneity. Twenty-eight observational studies were identified. All assessed AAM in women (combined *N =* 1,228,306); only 1 study additionally included men. In models without adjustment for adult adiposity, T2D/IGT risk was lower per year later AAM (relative risk [RR] = 0.91, 95% CI 0.89–0.93, *p <* 0.001, 11 estimates, *n =* 833,529, *I*^2^ = 85.4%) and higher for early versus later menarche (RR = 1.39, 95% CI 1.25–1.55, *p <* 0.001, 23 estimates, *n =* 1,185,444, *I*^2^ = 87.8%). Associations were weaker but still evident in models adjusted for adiposity (AAM: RR = 0.97 per year, 95% CI 0.95–0.98, *p <* 0.001, 12 estimates, *n =* 852,268, *I*^2^ = 51.8%; early menarche: RR = 1.19, 95% CI 1.11–1.28, *p <* 0.001, 21 estimates, *n =* 890,583, *I*^2^ = 68.1%). Associations were stronger among white than Asian women, and in populations with earlier average AAM. The estimated population attributable risk of T2D in white UK women due to early menarche unadjusted and adjusted for adiposity was 12.6% (95% CI 11.0–14.3) and 5.1% (95% CI 3.6–6.7), respectively. Findings in this study are limited by residual and unmeasured confounding, and self-reported AAM.

**Conclusions:**

Earlier AAM is consistently associated with higher T2D/IGT risk, independent of adiposity. More importantly, this research has identified that a substantial proportion of T2D in women is related to early menarche, which would be expected to increase in light of global secular trends towards earlier puberty timing. These findings highlight the need to identify the underlying mechanisms linking early menarche to T2D/IGT risk.

## Abstract 2

Tuck Seng Cheng and colleagues examine the literature on the association between puberty timing and Type 2 diabetes.

## Author summary

**Why was this study done?:**

- Secular trends towards earlier puberty timing have led to interest in its long-term disease consequences, particularly the association between early age at menarche in women and the development of type 2 diabetes.
- An earlier pooled analysis of the association between puberty timing and risk of type 2 diabetes was limited to findings adjusted for adulthood adiposity and included studies mainly among Western women.
- The present study aimed to evaluate whether puberty timing is associated with type 2 diabetes/impaired glucose tolerance, independent of adiposity.

**What did the researchers do and find?:**

- This systematic review identified 28 observational studies that analysed age at menarche among women and type 2 diabetes/impaired glucose tolerance; 1 study additionally included age at voice breaking in men.
- Meta-analysis showed that risk for type 2 diabetes and impaired glucose tolerance is higher among women with early than later menarche, independent of adiposity.
- The risk for type 2 diabetes and impaired glucose tolerance among women with early menarche is even higher in white than Asian women and in populations with younger average age at menarche.

**What do these findings mean?:**

- Girls who experience earlier menarche than their peers within and between populations have a higher risk for type 2 diabetes in adulthood.
- Preventive strategies that avoid early puberty timing might reduce future risk of type 2 diabetes.

## Introduction

Puberty is the transitional period from childhood to adulthood when physiological and physical changes relating to sexual maturation occur to attain fertility. The onset of puberty is indicated by the appearance of breast buds in girls, genital development in boys, and pubic hair growth in both sexes, as defined and assessed by the Tanner scale [1,2]. In the later period of puberty (at Tanner stage 3 or 4), girls experience first menstruation, namely menarche [3], and boys experience voice break [4]. Within populations, timing of puberty varies widely by sex and between individuals. Recently reported age at onset of puberty ranges from 8 to 13 years in girls and from 9 to 14 years in boys [5,6]. However, marked decreases in the age of puberty are reported worldwide, particularly for age at menarche (AAM) in women, which tends to be widely assessed in studies [5,7–9], and it has been postulated that these trends reflect decreases in childhood undernutrition and increases in childhood adiposity [3].

In light of these secular trends, puberty timing has been widely examined in relation to health outcomes, including type 2 diabetes (T2D), which is increasingly prevalent worldwide [10]. An earlier systematic review and meta-analysis showed that early menarche was associated with higher T2D risk [11]. That review identified 10 relevant publications (315,428 participants) dated until the end of 2013 and included only 2 studies in non-Western settings (both were from China) [11], which did not allow for comparisons between regions. There have been several very large Asian studies published subsequently [12,13]. More importantly, this previous meta-analysis analysed only effect estimates adjusted for body mass index (BMI) [11]. As BMI was invariably measured in adulthood, rather than in childhood, it may be considered as a mediator between puberty timing and T2D, rather than simply a confounder, although BMI, overweight, and obesity track from early childhood to adulthood [14,15]. Comparison of the associations between puberty timing and T2D with and without adjustment for adiposity would be informative. Furthermore, a recent study from China reported that the association between AAM and incident diabetes differed by year of birth, with a stronger association observed in women who were born in more recent decades [12]. Such potential effect modifications were not investigated in the previous meta-analysis [11].

Here, we describe a systematic review and meta-analysis to evaluate the association between puberty timing and T2D and/or impaired glucose tolerance (IGT), with and without adjustment for adiposity, in both women and men. We also assess study-design-related factors that could explain the heterogeneity between study estimates. Finally, we estimate the potential contribution of early menarche to the population burden of T2D.

## Methods

### Study inclusion criteria

Published papers were included in the present systematic review if they reported (i) any measure of puberty timing reported in childhood or adulthood (pubertal onset: age at breast or genital development or Tanner stage 2 pubic hair [1,2]; pubertal completion: AAM or age at voice breaking) and (ii) T2D/IGT assessed by fasting plasma glucose, oral glucose tolerance test, and/or glycated haemoglobin; self-reported by participants; or based on medical records/physician diagnosis. No restriction was given to the sex or geographical locations of studied populations, nor to the type of study design, whether observational or experimental.

### Exclusion criteria

We excluded studies that analysed populations with specific diseases such as breast cancer, polycystic ovary syndrome, Turner syndrome, premature adrenarche, and type 1 or 2 diabetes, as well as animal studies. Papers published without a full report available in English language were not excluded by our search terms; however, no such paper was considered potentially relevant on screening of titles and abstracts in English.

### Data sources and searches

We searched online databases (i.e., PubMed, Medline, and Embase) until 28 February 2019. The search terms were (i) terms or measures related to puberty timing (e.g., puberty, menarche, voice break, Tanner) and (ii) terms or measures related to diabetes (e.g., diabetes, glucose, insulin, glycated haemoglobin) and (iii) terms related to epidemiological studies (based on guidelines from the Scottish Intercollegiate Guidelines Network) [16]. Further details of the search strategy are shown in S1 Table. All identified papers were screened by title and abstract, and if considered potentially relevant, the full texts were read for inclusion decision. Any uncertainty about the eligibility of a particular study was resolved through discussion between authors (TSC and KKO). We also reviewed studies included in the previous systematic review [11] and the reference lists of our included papers to identify relevant papers. The present study was registered in the International Prospective Register of Systematic Reviews (PROSPERO registration number: CRD42019124353), and the protocol is available at: http://www.crd.york.ac.uk/PROSPERO/display_record.php?ID=CRD42019124353.

### Data extraction

Data from eligible studies for systematic review were extracted by one author (TSC); a 20% sample was independently extracted by a second author (RL), blinded to the original dataset, which was verified (100% agreement) by a third author (KKO).

Extracted information included first author, publication year, sample size, study population and ethnicity, year at enrolment, ages at puberty and outcome assessment, mean AAM, number of cases, definition of outcome, types of outcomes (prevalent or incident T2D/IGT cases), risk estimates with corresponding confidence intervals (CIs), definition of early puberty and its reference category, and variables controlled for in multivariable models. Specifically, for meta-analysis, we selected (i) risk estimates for T2D/IGT per year later AAM as a continuous variable (i.e., dose–response relationship) and (ii) risk estimates for T2D/IGT in the earlier AAM category compared to the middle or older AAM category (i.e., categorical relationship). We distinguished between estimates from models adjusted for potential confounders (but not adiposity) and estimates from models adjusted for an adiposity indicator (usually BMI or waist circumference; if available, estimates adjusted for both were preferentially extracted). If a study reported estimates for multiple outcomes, we prioritised the risk estimate for combined T2D/IGT, followed by T2D only and IGT only, and included the estimate for only 1 such outcome per study.

For those studies that reported risk estimates for T2D/IGT per year earlier (rather than later) AAM [17], we calculated the reciprocals to produce risk estimates per year later AAM. Similarly, for those studies that reported risk estimates for T2D/IGT in an older (rather than earlier) AAM category [12,18–21] compared to an earlier AAM category as the reference, we calculated the reciprocals to produce risk estimates in the earlier AAM category compared to the older AAM category as the reference. We considered odds ratios (ORs) and hazard ratios (HRs) to be similar estimates of the relative risk (RR) since findings were similar by these measures of association.

### Data synthesis and analysis

To summarise the association between AAM and T2D/IGT, we produced inverse-variance-weighted random-effects models, which allow for heterogeneity among individual study effect estimates. Estimates from models with and without adjustment for adiposity indicators were considered separately. Heterogeneity between studies was quantified by the inconsistency index (*I*^2^) (<50%, 50%–75%, and >75% indicated mild, moderate, and high heterogeneity, respectively). Potential sources of heterogeneity were evaluated using meta-regression analyses. Asymmetry was evaluated using visual inspection of funnel plots and Egger’s regression test. Sensitivity analyses by the trim-and-fill and leave-one-out methods were performed. Statistical analyses were performed using the “metafor” package in R software [22]. *p-*Values < 0.05 were considered to indicate statistical significance.

Based on the causal assumption that AAM affects T2D/IGT risk, which underlies the interpretation of population attributable risk as the proportion of preventable disease [23], the population attributable risk for T2D/IGT due to early menarche among British women was calculated using the formula $\frac{p(RR-1)}{p(RR-1)+1}$, where *p* is the prevalence of early menarche (defined as <12 years) in the large population-based UK Biobank study [24], and RR is the pooled risk estimate among white populations.

### Quality assessment

The Newcastle–Ottawa Quality Assessment Scale for cohort studies [25] was used to assess the quality of each study included in the systematic review. Criteria for each item in the assessment scale were defined according to the present research topic before study quality assessments were performed. For longitudinal studies of incident T2D/IGT and longitudinal studies that assessed puberty timing in adolescence and early adulthood and subsequent prevalent T2D/IGT, all 8 items were applied (maximum score of 9). For cross-sectional studies of prevalent T2D/IGT, only 6 items (maximum score of 7) were used (presence of T2D/IGT at baseline and follow-up duration were not relevant).

## Results

### Study characteristics

**Fig 1:**
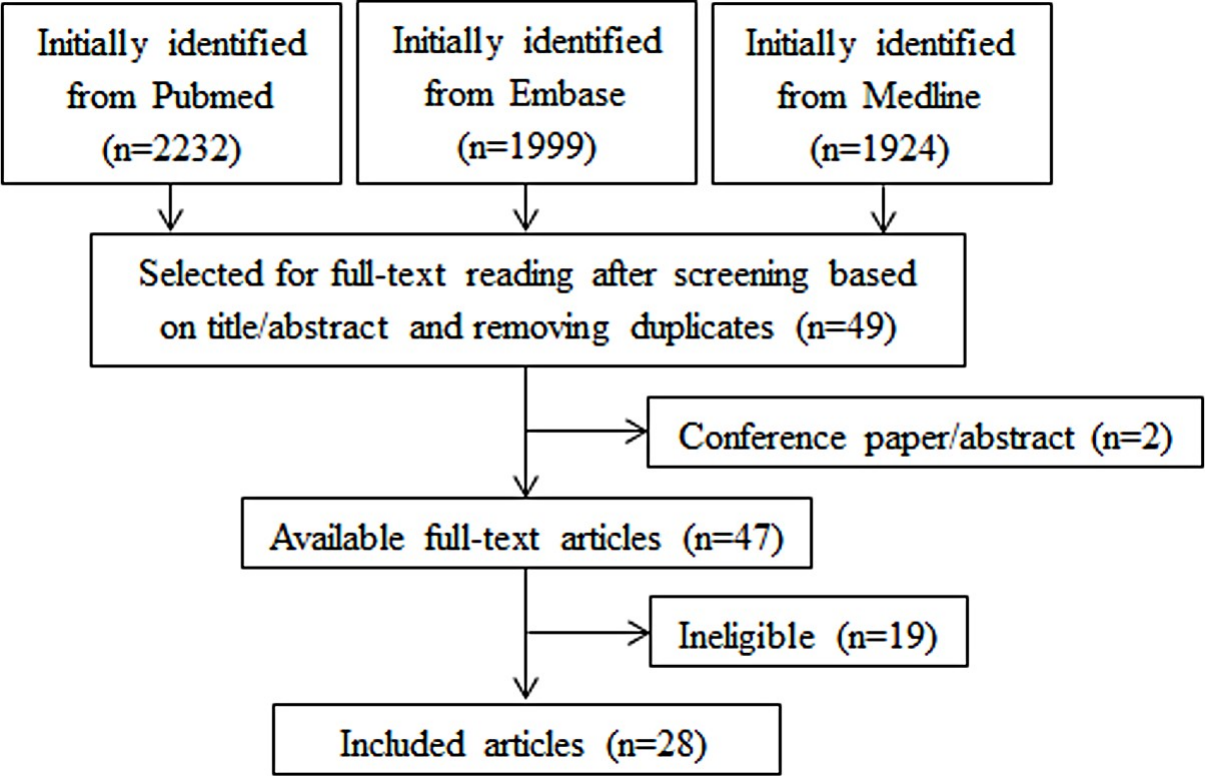
Flowchart of study selection.

Study selection is summarised in Fig 1. The search strategy identified 6,155 records. After screening based on titles and abstracts, and removing duplicates and non-relevant studies, 49 texts were selected for full-text reading, and finally 28 studies were deemed eligible for inclusion in the review. All 10 studies included in the previous review [11] and studies in the reference lists of included studies were found in the databases by our search strategy.

Tables 1 and 2 (and S2 and S3 Tables) show the characteristics of the included studies for the outcomes prevalent and incident cases of T2D/IGT, respectively. Of the 28 included studies, all assessed AAM in women (combined *N =* 1,228,306), and only 1 additionally analysed age at voice breaking in men [24]. Data on puberty timing was collected during middle to late adulthood in most studies (mean age ranging from 35 to 70 years), except during adolescence in 1 study [26] and in early adulthood (age < 25 years) in 2 studies [17,27]. All were observational studies, and 1 additionally included a Mendelian randomisation analysis [13]. Nine studies were conducted among white individuals [18,19,24,26–31], 13 studies among Asian individuals (Chinese, Bangladeshi, Korean, and Japanese) [12,13,20,21,32–40], and 6 studies among multi-ethnic populations (white, Hispanic, Asian, African-American, and Latino) [17,41–45]. Fourteen studies examined prevalent T2D [13,18,19,24,26,27,30,34,36–39,43,45], 2 prevalent IGT [21,32], 3 prevalent T2D and IGT [28,33,35], 8 incident T2D [12,17,20,29,31,40,41,44], and 1 prevalent and incident T2D [42]. The definitions of T2D and IGT varied across studies, and 4 studies excluded participants with potential type 1 diabetes based on age at diagnosis [24,26,42,43]. Adiposity indicators were adjusted for in 25 studies and were mostly BMI alone (*n =* 19) [17–21,26–31,35,37,39–41,43–45], followed by both BMI and waist circumference (*n =* 4) [12,34,36,42], waist circumference alone (*n =* 1) [32], and body composition (*n =* 1) [24]. Early menarche was defined as AAM < 12 years in 9 studies [17,24,29–31,38,41–43] and <14 years in 13 studies [12,18,20,21,32–37,39,40,44], while the reference category of AAM was defined as AAM ≥ 12 years in 12 studies [24,29–31,33,35–37,41–44] and ≥14 years in 10 studies [12,17,18,20,21,32,34,38–40]. Furthermore, the reference category of AAM was the middle category in 12 studies [24,29–31,34–36,39,41–44] and the oldest category in 10 studies [12,17,18,20,21,32,33,37,38,40]. Most studies (*n =* 18) tested the association of AAM with T2D/IGT risk using logistic regression models and reported ORs [13,18,19,21,24,27,30,32–42], while 6 studies used Cox proportional-hazards models and reported HRs [12,17,20,26,29,44], and 4 studies reported RRs using Poisson regression [28,43], log binomial regression [45], or generalised linear modelling [31].

**Table 1:** Summary of eligible studies of prevalent diabetes/IGT.

First author, year [reference]	*N* total (*N* cases)	Study; ethnicity	Year at enrolment	AAM (y)^a^	Age at outcome assessment (y)^a^	Outcome: Definition	Measure of association	Adiposity-unadjusted RR (95% CI)	Adiposity-adjusted RR (95% CI)	Adiposity covariate
Cooper, 2000 [27]	668 (49)	Menstruation and Reproductive History; white	1934–1939	12.4 (range: 8–18)	73 (range: 63–81)	Diabetes: Self-reported physician diagnosis	OR	—	1.1 (0.9, 1.3) per year	BMI
Saquib, 2005 [18]	997 (125)	Rancho Bernardo; white	1984–1987	<12: 14.5%; 12–15: 78.9%; ≥16: 6.6%	69.5 ± 9.3 (range: 50–92)	Diabetes: OGTT, physician diagnosis, or anti-diabetic medication	OR	—	2.27 (0.62, 9.09)^b^, *p =* 0.21, <12 vs. ≥16 (Ref)	BMI
Heys, 2007 [32]	7,108 (—)	Guangzhou Biobank; Chinese	2003–2004	15.4 ± 2.1 (range: 8–25)	64.0 ± 6.0 (range: 50–94)	IGT: Fasting glucose or anti-diabetic medication	OR	1.40 (1.15, 1.71), <12.5 vs. ≥14.5 (Ref)	1.33 (1.08, 1.63), <12.5 vs. ≥14.5 (Ref)	WC
Lakshman, 2008 [19]	13,308 (734)	EPIC-Norfolk; mainly white	1993–1997	13.0 ± 1.6	40–75	Diabetes: Self-reported physician diagnosis or anti-diabetic medication	OR	0.91 (0.87, 0.96) per year, *p <* 0.001; 1.52 (1.18, 1.96)^b^, *p-*trend = 0.001, 8–11 vs. 15–18 (Ref)	0.98 (0.93, 1.03) per year, *p =* 0.4	BMI
Akter, 2012 [33]	1,423 (—)	Gabindagonj Upazilla; Bangladeshi	2009–2010	Unknown	40.9 to 42.7 (by AAM group)	Diabetes: Physician diagnosis or anti-diabetic medication IGT: fasting glucose	OR	0.65 (0.46, 0.93), *p-*trend = 0.02, <12 vs. >13–16 (Ref)	—	—
Dreyfus, 2012 [42]	8,491 (990)	ARIC; white, African-American	1987–1989	12.9 ± 1.6	50.6 ± 9.3	Diabetes: Fasting/non-fasting glucose, self-reported physician-diagnosis, or anti-diabetic medication	OR	1.37 (1.12, 1.68), *p <* 0.05, 8–11 vs. 13 (Ref)	1.20 (0.97, 1.48), *p* > 0.05, 8–11 vs. 13 (Ref)	BMI, WC
Pierce, 2012 [26]	1,632 (26)	NSHD; white	1946	13.2 (range: 8.5–19.5)	31, 36, 43, 53	Diabetes: Ever treated	HR	0.72 (0.52, 0.99) per year, *p =* 0.05	0.86 (0.63, 1.18) per year, *p =* 0.5	BMI
Stockl, 2012 [28]	1,503 (366)	KORA; white	2006–2008	13.5 ± 1.6	Range: 25–74	Diabetes: OGTT, physician diagnosis, or anti-diabetic medication IGT: OGTT	RR	0.88 (0.83, 0.94) per year, *p <* 0.001	0.89 (0.83, 0.95) per year, *p <* 0.001	BMI
Qiu, 2013 [34]	3,304 (738)	Chinese	2011–2012	Median: 16 (IQR: 15–18)	59 (range: 37–92)	Diabetes: OGTT, physician diagnosis, or anti-diabetic medication	OR	0.94 (0.70, 1.26), *p =* 0.682, 9–14 vs. 16 (Ref)	0.90 (0.66, 1.21), *p =* 0.479, 9–14 vs. 16 (Ref)	BMI, WC
Mueller, 2014 [43]	8,075 (1,335)	ELSA-Brasil; white and black Brazilian	2008–2010	12.7 ± 1.7	52.0 ± 8.8 (range: 35–74)	Diabetes: OGTT, HbA1c, physician diagnosis, or anti-diabetic medication	RR	1.34 (1.14, 1.57), <11 vs. 13–14 (Ref)	1.26 (1.07, 1.49), <11 vs. 13–14 (Ref)	BMI
Baek, 2015 [35]	2,039 (905)	Sungkyunkwan University; Korean	2012–2013	14.6 ± 1.6	48.9 ± 3.5 (range: 44–56)	Diabetes: OGTT, HbA1c, physician diagnosis, or anti-diabetic medication IGT: fasting glucose or HbA1c	OR	1.85 (1.28, 2.66), *p =* 0.001, <13 vs. 13–16 (Ref)	1.66 (1.14, 2.41), *p =* 0.008, <13 vs. 13–16 (Ref)	BMI
Day, 2015 [24]	250,037 (4,836)	UK Biobank; white	2006–2010	13.0 ± 1.6 (range: 8–19)	56.52 ± 8.09 (range: 40–69)	Diabetes: Self-reported physician diagnosis	OR	0.87 (0.85, 0.88) per year, *p <* 0.001; 1.76 (1.62, 1.91), *p <* 0.001, 8–11 vs. 13–14 (Ref)	0.94 (0.92, 0.96) per year, *p <* 0.001; 1.25 (1.15, 1.36), *p <* 0.001, 8–11 vs. 13–14 (Ref)	Body comp.
Hwang, 2015 [36]	3,254 (—)	KNHANES IV; Korean	2007–2009	15.67	64.1 (range: 50–85)	Diabetes: Self-reported physician diagnosis (including type 1 and 2)	OR	1.86 (1.07, 3.23), *p <* 0.05, 10–12 vs. 13–15 (Ref)	1.82 (1.03, 3.23), *p <* 0.05, 10–12 vs. 13–15 (Ref)	BMI, WC
Lim, 2015 [37]	4,326 (119)	KNHANES IV; Korean	2007–2009	13.0 to 14.3 (by age group)	Range: 20–50	Diabetes: Fasting glucose, self-reported physician diagnosis, or anti-diabetic medication	OR	3.61 (1.90, 6.88), *p <* 0.05, <12 vs. ≥12 (Ref)	2.52 (1.29, 4.94), *p <* 0.05, <12 vs. ≥12 (Ref)	BMI
Cao, 2016 [21]	1,625 (—)	Changsha Women’s Health Screening Program; Chinese	2011–2014	—	60.45 ± 8.19 (range: 40–75)	IGT: Fasting glucose	OR	—	0.83 (0.62, 1.10)^b^, 11–13 vs. 16–20 (Ref)	BMI
Won, 2016 [38]	12,336 (—)	KNHANES; Korean	2010–2013	14.6	45.7	Diabetes: Self-reported physician diagnosis	OR	1.72 (0.94, 3.15), *p =* 0.077, <11 vs. ≥17 (Ref)	—	—
Yang, 2016 [39]	16,114 (832)	Jinchang Cohort; Chinese	2011–2013	14.8 ± 2.0	45.8 ± 11.8	Diabetes: Fasting glucose or anti-diabetic medication	OR	1.60 (1.16, 2.22), *p <* 0.05, ≤12 vs. 15–16 (Ref)	1.44 (1.02, 2.03), *p <* 0.05, ≤12 vs. 15–16 (Ref)	BMI
Au Yeung, 2017 [13]	12,484 (—)	Guangzhou Biobank; Chinese	2003–2008	14.3 to 15.9 (by age group)	≥50	Diabetes: Fasting glucose or anti-diabetic medication	OR	0.92 (0.89, 0.95) per year	—	—
Farahmand, 2017 [30]	4,952 (187)	Tehran Lipid and Glucose; white	1998	13.3 ± 1.5	28.1 to 36.9 (by AAM group)	Diabetes: OGTT	OR	2.70 (1.40, 5.20), <11 vs. 13–14 (Ref)	3.28 (1.50, 7.10), <11 vs. 13–14 (Ref)	BMI
Petersohn, 2019 [45]	30,626 (2,328)	Mexican National Health survey; Mexican	1999–2000	13	37 to 45 (by AAM group)	Diabetes: Self-reported physician diagnosis or OGTT	RR	—	0.95 (0.83, 0.98) per year, *p <* 0.001	BMI

^a^Mean or mean ± SD unless otherwise indicated.

^b^Result was computed as the reciprocal of the risk estimate for the highest category.

AAM, age at menarche; BMI, body mass index; comp., composition; HR, hazard ratio; IGT, impaired glucose tolerance; OGTT, oral glucose tolerance test; OR, odds ratio; RR, relative risk; WC, waist circumference; y, years.

**Table 2:** Summary of eligible studies of incident diabetes/IGT.

First author, year [reference]	Total *N* (*N* cases)	Study; ethnicity	Year at enrolment	AAM (y)^a^	Age at outcome assessment or duration of follow-up (y)^a^	Outcome: Definition	Measure of association	Adiposity-unadjusted RR (95% CI)	Adiposity-adjusted RR (95% CI)	Adiposity covariate
He, 2010 [41]	101,415 (7,963)	Nurses’ Health; multi-ethnic	1980	—	63.5	Diabetes: OGTT, ≥1 diabetes symptom or anti-diabetic medication	OR	0.94 (0.92, 0.95) per year, *p* < 0.05; 1.21 (1.13, 1.31), *p* < 0.001, ≤11 vs. 13 (Ref)	0.99 (0.97, 1.01) per year, *p* > 0.05; 1.02 (0.95, 1.10), *p =* 0.42, ≤11 vs. 13 (Ref)	BMI
	100,547 (2,739)	Nurses’ Health II; multi-ethnic	1991	—	47.4			0.88 (0.86, 0.91) per year, *p <* 0.05; 1.50 (1.34, 1.69), *p <* 0.001, ≤11 vs. 13 (Ref)	0.97 (0.94, 1.00) per year, *p* > 0.05; 1.15 (1.02, 1.29), *p =* 0.19, ≤11 vs. 13 (Ref)	BMI
Conway, 2012 [20]	69,385 (1,831)	Shanghai Women’s Health; Chinese	1997–2000	—	60.1 ± 2.0	Diabetes: OGTT or anti-diabetic medication	HR	0.95 (0.92, 0.98) per year; 1.35 (1.14, 1.59)^b^, 8–13 vs. 17–26 (Ref)	0.98 (0.95, 1.01) per year; 1.14 (0.95, 1.33)^b^, 8–13 vs. 17–26 (Ref)	BMI
Dreyfus, 2012 [42]	7,501 (755)	ARIC; white, African-American	1987–1989	12.9 ± 1.6	56.8 ± 8.0	Diabetes: fasting/non-fasting glucose, self-reported physician-diagnosis, or anti-diabetic medication	OR	1.27 (1.02, 1.58), *p <* 0.05, 8–11 vs. 13 (Ref)	1.18 (0.95, 1.47), *p* > 0.05, 8–11 vs. 13 (Ref)	—
Elks, 2013 [29]	10,903 (4,242)	EPIC-InterAct; white	1991	13.14 ± 1.58	52	Diabetes: Health-record-confirmed self-reported physician diagnosis	HR	0.89 (0.86, 0.93) per year, *p <* 0.001; 1.70 (1.48, 1.94), *p <* 0.001, 8–11 vs. 13 (Ref)	0.96 (0.91, 1.01) per year, *p =* 0.11; 1.42 (1.18, 1.71), *p <* 0.001, 8–11 vs. 13 (Ref)	BMI
Dreyfus, 2015 [17]	1,970 (271)	CARDIA; white, African-American	1985	12.6 ± 1.5 (range: 8–16)	50 (range: 42–59)	Diabetes: OGTT or anti-diabetic medication	HR	0.93 (0.86, 1.00) per year^c^, 1.61 (1.09, 2.37), 8–11 vs. 14–17 (Ref)	0.90 (0.86, 0.94) per year^c^, 1.33 (0.90, 1.96), 8–11 vs. 14–17 (Ref)	BMI
LeBlanc, 2017 [44]	124,379 (11,262)	Women’s Health Initiative; multi-ethnic	1993–1998	—	Follow-up: 12.2 ± 4.2	Diabetes: Self-reported diagnosis or anti-diabetic medication	HR	1.14 (1.08, 1.20), *p <* 0.001, <12 vs. 12 (Ref)	1.01 (0.95, 1.06), *p =* 0.89, <12 vs. 12 (Ref)	BMI
Yang, 2018 [12]	270,345 (5,391)	China Kadoorie Biobank; Chinese	2004–2008	15.4 ± 1.9	Follow-up: 7	Diabetes: Health records	HR	0.96 (0.94, 0.97) per year, *p-*trend < 0.001; 1.33 (1.24, 1.44)^b^, 13 vs. ≥18 (Ref)	0.98 (0.97, 1.00) per year	BMI, WC
Pandeya, 2018 [31]	126,721 (4,073)	InterLACE; mainly white	1985–2009	13.1 (range: 8–20)	56.1 ± 11.4	Diabetes: Self-reported physician diagnosis or health records	RR	1.63 (1.40, 1.89), ≤10 vs. 13 (Ref)	1.18 (1.02, 1.37), ≤10 vs. 13 (Ref)	BMI
Nanri, 2019 [40]	37,511 (513)	Japan Public Health Center-based Prospective Study; Japanese	1990, 1993	14.7 ± 1.9	Follow-up: 10	Diabetes: Health-record-confirmed self-reported physician diagnosis	OR	1.09 (0.83, 1.43)^b^, *p-*trend = 0.44, ≤13 vs. ≥16 (Ref)	1.01 (0.76, 1.33)^b^, *p-*trend = 0.82, ≤13 vs. ≥16 (Ref)	BMI

^a^Mean or mean ± SD unless otherwise indicated.

^b^Result was computed as the reciprocal of the risk estimate for the highest category.

^c^Result was computed as the reciprocal of the risk estimate per year earlier AAM.

AAM, age at menarche; BMI, body mass index; HR, hazards ratio; IGT, impaired glucose tolerance; OGTT, oral glucose tolerance test; OR, odds ratio; RR, relative risk; WC, waist circumference; y, years.

For models without adjustment for adiposity, most studies (*n =* 20/24) reported a statistically significant association with higher T2D/IGT risk for earlier menarche [12,13,17,19,20,24,26,28–32,35–37,39,41–44] or earlier voice breaking [24]; only 3 reported no association [34,38,40], and 1 study reported that earlier menarche was associated with lower T2D/IGT risk [33]. For models with adjustment for adiposity, some studies (*n =* 11/24) reported a statistically significant association with higher T2D/IGT risk for earlier menarche [12,24,28,30–32,35–37,39,43] or earlier voice breaking [24], but other studies did not (*n =* 11) [17–21,26,27,34,40,42,44], and 2 studies reported inconsistent findings between dose–response and categorical AAM models [29] or between sub-cohorts [41].

### Quality assessment

More than half of studies of prevalent T2D/IGT (*n =* 11 studies) scored 6/7, followed by 5/7 (*n =* 4), 7/7 (*n =* 3), and 5/9 (*n =* 2) (S4 Table). Longitudinal studies of incident T2D/IGT were rated 9/9 (*n =* 5) or 8/9 (*n =* 4) (S5 Table).

### Meta-analysis results

All 28 studies on AAM and T2D/IGT in women were included in the meta-analysis. Similar findings were observed for pooled estimates for T2D only and IGT only (S1 and S2 Figs). To maximise power, we therefore prioritised risk estimates for combined T2D/IGT (3 studies), followed by T2D only (23 studies) and IGT only (2 studies).

**Fig 2:**
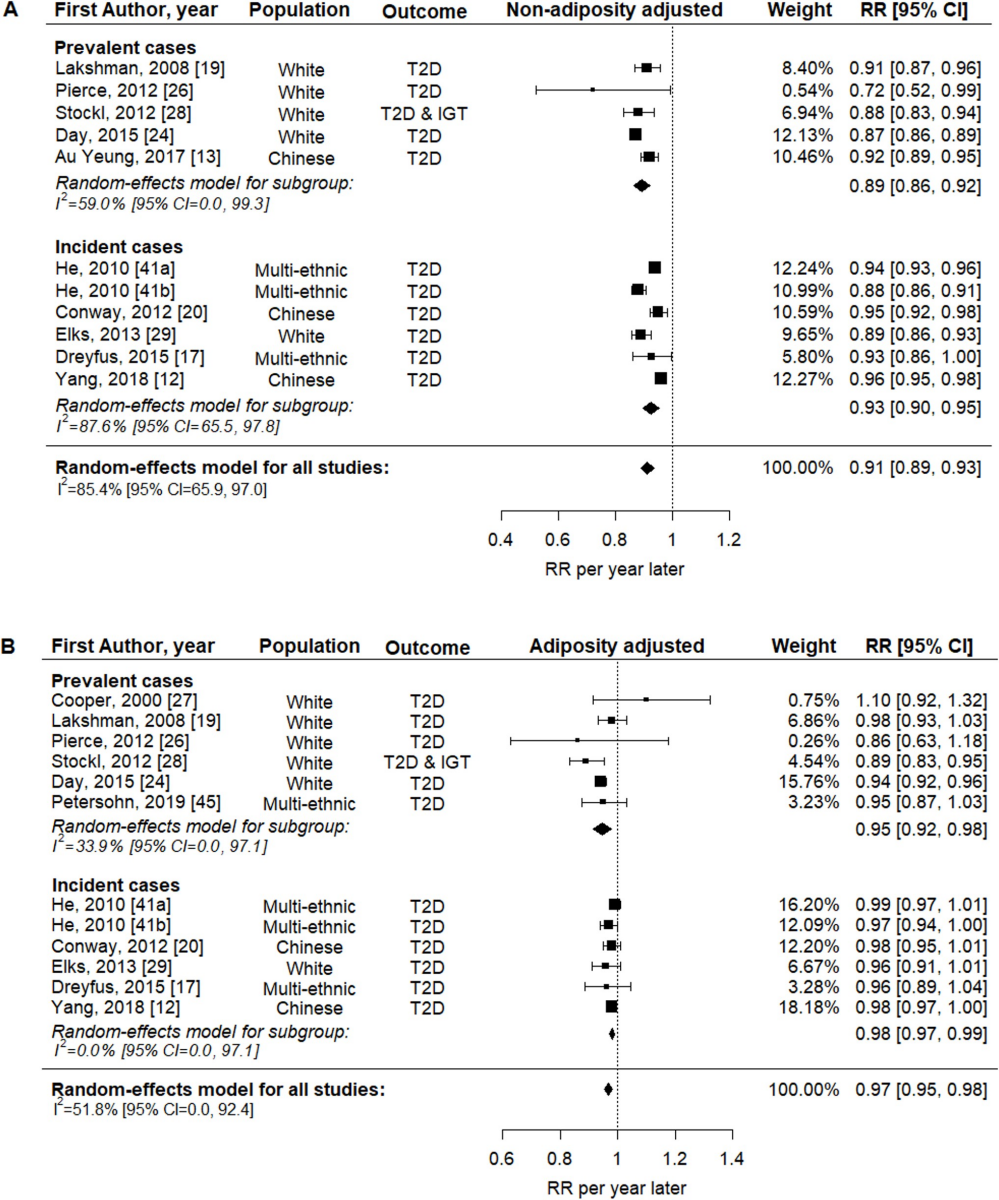
Forest plots of the association between AAM (continuous variable) and T2D/IGT, without and with adjustment for adiposity. (A) Without and (B) with adjustment for adiposity. Two cohort studies in He, 2010 [41]. AAM, age at menarche; IGT, impaired glucose tolerance; RR, relative risk; T2D, type 2 diabetes.

Fig 2 shows the association between continuous AAM and T2D/IGT. From models without adjustment for adult adiposity, pooled analysis of 11 estimates from 10 studies showed that later AAM was associated with lower T2D/IGT risk (RR = 0.91 per year, 95% CI 0.89–0.93, *p <* 0.001, *n =* 833,529; Fig 2A). This association was weaker but still evident in models with adjustment for adiposity (pooled analysis of 12 estimates from 11 studies: RR = 0.97 per year, 95% CI 0.95–0.98, *p <* 0.001, *n =* 852,268; Fig 2B). Similar findings were obtained in subgroup analyses by prevalent or incident T2D/IGT (Fig 2). Heterogeneity between studies was high in estimates without adjustment for adiposity (*I*^2^ = 85.4%) and moderate in estimates with adjustment for adiposity (*I*^2^ = 51.8%).

**Fig 3:**
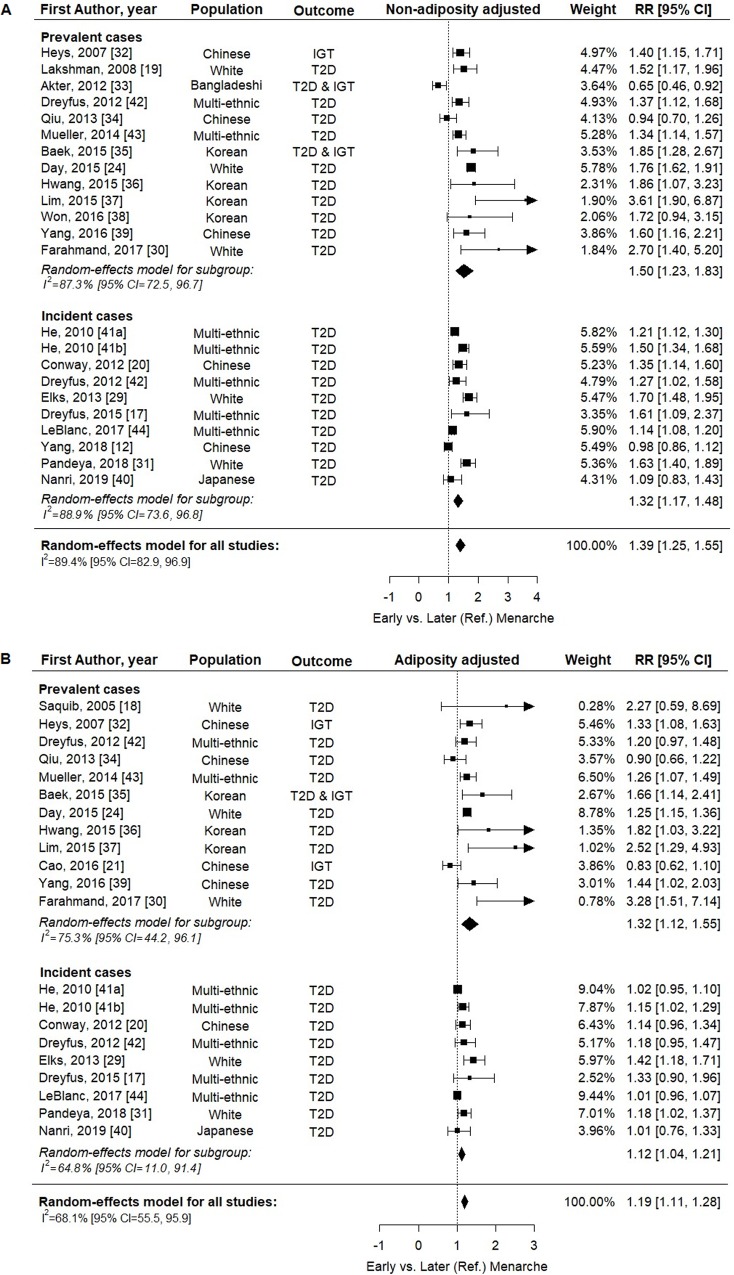
Forest plots of the association between early versus later menarche and T2D/IGT, without and with adjustment for adiposity. (A) Without and (B) with adjustment for adiposity. Two cohort studies in He, 2010 [41]. AAM, age at menarche; IGT, impaired glucose tolerance; RR, relative risk; T2D, type 2 diabetes.

Fig 3 shows the association between categorical early versus later menarche and T2D/IGT. From models without adjustment for adult adiposity, pooled analysis of 23 estimates from 21 studies showed that early menarche was associated with higher T2D/IGT risk (RR = 1.39, 95% CI 1.25–1.55, *p <* 0.001, *n =* 1,185,444; Fig 3A). This association was weaker but still evident in models with adjustment for adiposity (pooled analysis of 21 estimates from 19 studies: RR = 1.19, 95% CI 1.11–1.28, *p <* 0.001, *n =* 890,583; Fig 3B). Similar findings were obtained in subgroup analyses by prevalent or incident T2D/IGT (Fig 3). Heterogeneity between studies was high in estimates without adjustment for adiposity (*I*^2^ = 87.8%) and moderate in estimates with adjustment for adiposity (*I*^2^ = 68.1%).

### Meta-regression results

Table 3 shows results of univariable meta-regression and pooled RRs by subgroups of studies. Heterogeneity between studies was partially explained by study-level differences in ethnicity and average AAM. The T2D/IGT risk associated with earlier menarche (both continuous and categorical) was even higher among studies of white individuals than that among Asian individuals, and was also higher among populations with younger than older average AAM. Year of enrolment, age at outcome assessment, number of variables adjusted for, age cutoff used to define early menarche and the reference category, and measure of association (OR, HR, or RR) did not explain the heterogeneity between study estimates (S6 Table).

**Table 3:** Univariable meta-regression results (*R*^2^ and *p*-values) and pooled RR for diabetes and impaired glucose tolerance in study subgroups.

Factor and subgroup	RR per year later age at menarche								Early versus later (Ref) menarche							
	Not adiposity adjusted				Adiposity adjusted				Not adiposity adjusted				Adiposity adjusted			
	*N*	RR (95% CI)	*p*-Value^a^	*R*^2^ (%)	*N*	RR (95% CI)	*p*-Value^a^	*R*^2^ (%)	*N*	RR (95% CI)	*p*-Value^a^	*R*^2^ (%)	*N*	RR (95% CI)	*p*-Value^a^	*R*^2^ (%)
Ethnicity				53.7				99.63				37.9				16.3
Asian	3	0.95 (0.92, 0.97)			2	0.98 (0.97, 0.99)			11	1.33 (1.06, 1.69)			9	1.23 (1.02, 1.49)		
White	5	0.88 (0.86, 0.90)	0.002		6	0.95 (0.92, 0.98)	0.001		5	1.72 (1.61, 1.83)	0.013		5	1.27 (1.18, 1.36)	0.290	
Multi-ethnic	3	0.91 (0.87, 0.96)	0.154		4	0.98 (0.96, 1.00)	0.871		7	1.30 (1.18, 1.42)	0.743		7	1.11 (1.02, 1.20)	0.472	
Study average AAM, years^b^				33.3				0				38				18.9
<13.5	5	0.89 (0.86, 0.91)			6	0.96 (0.93, 0.98)			8	1.59 (1.45, 1.75)			7	1.26 (1.19, 1.34)		
≥13.5	2	0.92 (0.85, 1.01)	0.154		2	0.94 (0.86, 1.03)	0.719		8	1.36 (1.17, 1.58)	0.036		6	1.27 (1.03, 1.55)	0.820	
*p*-Value for linear trend^c^			<0.001				0.400				0.014				0.677	

*N* is the number of estimates; *R*^2^ (%) is the percent of heterogeneity explained.

^a^The reference category in meta-regression models is the first subgroup in each factor.

^b^Studies that did not report the information were excluded.

^c^Using study average AAM as a continuous variable.

AAM, age at menarche; RR, relative risk.

### Sensitivity analyses

S3 Fig shows some asymmetry in funnel plots for studies on the association between categorical early menarche and T2D/IGT, which was statistically significant only for the studies on early versus later menarche and T2D/IGT with adjustment for adiposity (Egger’s test, *p <* 0.001). The predominant source of asymmetry was the small studies, whereas the findings of the larger studies appeared to be consistent with the overall estimates.

Sensitivity analyses were performed to account for this asymmetry. S4 Fig shows the predicted missing studies using the trim-and-fill method. When the predicted missing studies were added to the meta-analyses, the associations between earlier continuous AAM (adiposity-unadjusted RR = 0.91 per year, 95% CI 0.89–0.94; adiposity-adjusted RR = 0.97 per year, 95% CI 0.95–0.98) and categorical AAM (adiposity-unadjusted RR = 1.35, 95% CI 1.21–1.49; adiposity-adjusted RR = 1.15, 95% CI 1.06–1.24) and higher T2D/IGT risk remained similar.

S5 Fig shows the results of leave-one-out analyses. When 1 of the study estimates was iteratively removed from the meta-analysis, the pooled estimates remained nearly unchanged for associations between earlier AAM (continuous and categorical) and higher T2D/IGT risk, with or without adjustment for adiposity.

### Contribution of early menarche to the burden T2D

In light of the observed higher T2D/IGT risk associated with early menarche in white than Asian individuals, and the availability of data from UK Biobank—a very large population-based study of predominantly white adults—we used the pooled RR in white populations and the prevalence of early menarche in white women in UK Biobank to estimate the current maximum contribution of early menarche to the burden of T2D. The estimated population attributable risk for T2D/IGT due to early menarche (<12 years) among white British women (prevalence 20.15% in UK Biobank) unadjusted for adult adiposity was 12.6% (95% CI 11.0%–14.3%, *p <* 0.001) and adjusted for adult adiposity was 5.1% (95% CI 3.6%–6.7%, *p <* 0.001).

## Discussion

The present meta-analysis of observational studies showed that earlier AAM is associated with higher T2D/IGT risk; this association is weaker but still evident after adjustment for adult adiposity. Study quality was in general high, and, despite evidence of asymmetry due to small study effects in 1 of the 4 models, similar findings were obtained in sensitivity analyses that considered predicted missing studies. Heterogeneity between studies was high and was partially explained by study differences in ethnicity and average AAM, with stronger associations in white women and in study populations with lower average AAM. Assuming a causal relationship [23], a significant proportion of T2D/IGT among white British women may be attributable to early menarche (before age 12 years). We found a paucity of studies on puberty timing and T2D/IGT in men.

Our meta-analysis findings are consistent with a previous review [11], which reported associations of younger AAM and early menarche with higher T2D risk with adjustment for adiposity, but we (i) included a larger number of studies (19 versus 10) and women (890,583 versus 315,428), (ii) distinguished between findings unadjusted and adjusted for adiposity, and (iii) identified reasons for heterogeneity. While the previous meta-analysis [11] found an association of early menarche with higher T2D risk in Europe and the United States, we included more Asian studies and demonstrated that this association was also apparent in Asian individuals, although weaker than in white individuals, possibly due to their later average AAM. One study in China reported higher HRs for incident diabetes associated with younger AAM in women born in the 1960s–1970s than in the 1950s and 1920s–1940s, consistent with the decreased mean AAM over time, from 16.2 years in the 1920s–1940s to 14.7 years in the 1960s–1970s [12]. Hence, in light of worldwide secular trends towards lower average AAM [5,7–9], not only are more women moving into the high-risk group (early menarche), but also the magnitude of elevated risk in this group appears to be increasing.

The mechanisms that underlie the association between earlier AAM and higher T2D/IGT risk are unclear. Rapid postnatal weight gain [46] and childhood obesity [47,48] may precede early menarche, but also early menarche may promote adulthood obesity [49], and consequently increase T2D risk [3,50,51]. Hence, adiposity may be considered as both a partial confounder and partial mediator. However, our meta-analysis found that the association between earlier menarche and higher T2D/IGT risk remained, though attenuated, after accounting for the potential confounding and mediating effects of adiposity, suggesting that there may be other adiposity-independent underlying mechanisms. It has also been hypothesized that early menarche is a function of sex hormone exposure, such as higher levels of estradiol [52,53] and lower sex-hormone-binding globulin concentrations [54], in women, which may affect glycaemic regulation and increase risk of diabetes [55–57]. Nonetheless, hormone replacement therapy, predominantly with estrogen, was shown to reduce the incidence of diabetes [58]. Estrogen may have various effects on different parts of the body including brain, adipose tissue, breast, endometrium, and endothelium, probably mediated by different estrogen receptors [59].

We acknowledge several limitations of our study. We could not directly test or quantify the attenuation in the association when adjusting for adiposity, because the studies that contributed adjusted and unadjusted estimates were largely but not completely overlapping. All estimates were from observational studies, and thus residual confounding may exist. AAM was self-reported and was mainly recalled during adulthood, which may affect its accuracy; however, moderate correlations between prospective and recalled AAM several decades later have been reported [60,61]. Average AAM and cutoffs for early menarche and the reference category also varied across studies, and these were considered as sources of heterogeneity between study estimates. Some asymmetry was detected, especially for the adiposity-adjusted association between categorical early menarche and T2D/IGT, possibly indicating a bias towards reporting positive findings; however, this potential bias appeared to affect only small studies, and our sensitivity analyses were reassuring. Selection bias may exist due to the inclusion of only papers with full reports in English. We did not find any potentially relevant papers in other languages during screening of titles and abstracts in English, and our systematic review included many studies conducted in non-English-speaking populations; however, it is possible that other non-English studies are identifiable only in other publication databases. The subgroup analyses by study average AAM were limited to studies that reported this value. Although we examined relationships between both continuous and categorical AAM and T2D/IGT risk, we were unable to examine if there was any threshold of AAM that indicates higher risk of T2D/IGT, as was indicated by 1 large study [29]. Finally, we found only 1 study of puberty timing and T2D/IGT in men, likely because measures of puberty timing in men are not included in most studies. The 1 identified study was very large (*n =* 197,714) and reported a statistically robust association between relatively younger (versus about average) voice breaking and T2D in white men (adiposity-unadjusted RR = 1.44, 95% CI 1.30–1.59, *p <* 0.001; adiposity-adjusted RR = 1.24, 95% CI 1.11–1.37, *p <* 0.001) [24]. However, more such studies are needed, especially in non-white men, to understand whether the association could vary by population, as observed for women.

In conclusion, this systematic review and meta-analysis of observational studies showed that earlier AAM is consistently associated with higher T2D/IGT risk, independent of adiposity. This association is stronger among white individuals and populations with younger average AAM. We estimated that a substantial proportion of T2D cases in UK women was related to early menarche, and we would expect this proportion to increase in light of global secular trends towards earlier puberty timing. These findings warrant further studies to identify potential underlying mechanisms linking early menarche to future T2D/IGT risk.

## Supporting information

S1 PRISMA Checklist (DOC)

S1 Fig Forest plots of the association of AAM (as a continuous variable) with T2D or IGT, by prevalent or incident T2D/IGT. (A) Without and (B) with adjustment for adiposity.
(TIF)

S2 Fig Forest plots of the association of early versus later menarche with T2D or IGT, by prevalent or incident T2D/IGT. (A) Without and (B) with adjustment for adiposity.
(TIF)

S3 Fig Funnel plots and Egger’s tests for studies using AAM (continuous variable) and early menarche, with and without adjustment for adiposity. (A) AAM (continuous variable) and (B) early menarche.
(TIF)

S4 Fig Funnel plots with missing studies, with and without adjustment for adiposity, identified by the trim-and-fill method. (A) AAM (continuous variable) and (B) early menarche. Open circles indicate filled missing studies.
(TIF)

S5 Fig Forest plots of leave-one-out analysis, where each estimate in studies using AAM (continuous variable) and early menarche, with and without adjustment for adiposity, was iteratively removed. (A) AAM (continuous variable) and (B) early menarche.
(TIF)

S1 Table Search strategy in different databases. (DOCX)

S2 Table Summary of eligible studies for prevalent diabetes and/or IGT. (DOCX)

S3 Table Summary of eligible studies for incident diabetes and/or IGT. (DOCX)

S4 Table Quality of eligible studies for prevalent diabetes/IGT assessed by the Newcastle–Ottawa Quality Assessment Scale for cohort studies. (DOCX)

S5 Table Quality of eligible studies for incident diabetes/IGT assessed by the Newcastle–Ottawa Quality Assessment Scale for cohort studies. (DOCX)

S6 Table Univariable meta-regression results and pooled RR for diabetes and IGT in study subgroups. (DOCX)

## Abbreviations

AAM: age at menarche
BMI: body mass index
HR: hazard ratio
IGT: impaired glucose tolerance
OR: odds ratio
RR: relative risk
T2D: type 2 diabetes
