# Supplementary figures and images for: Association of puberty timing with type 2 diabetes: A systematic review and meta-analysis
Source: PLoS Med. 2020 Jan 6;17(1):e1003017. doi: 10.1371/journal.pmed.1003017 (PMC6944335; doi:10.1371/journal.pmed.1003017)

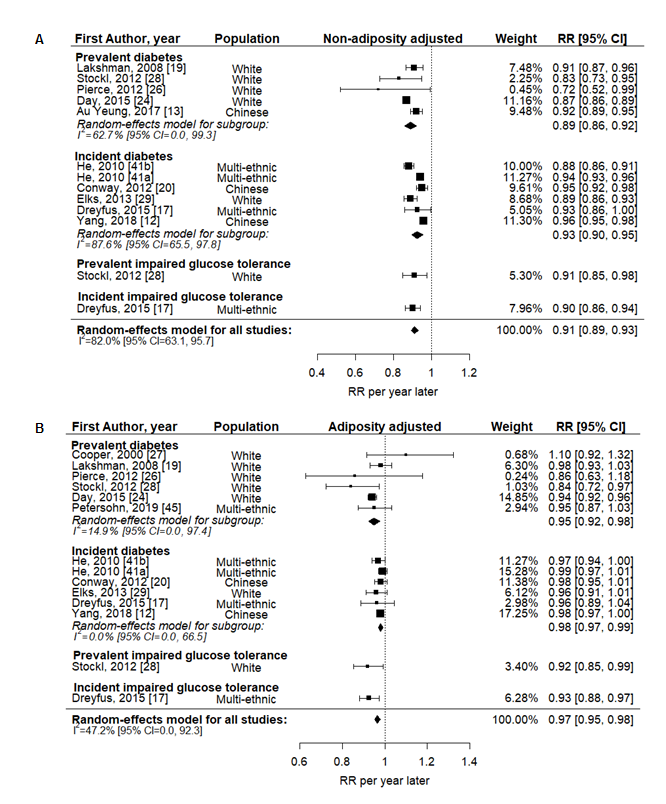

Supplement: S1 Fig — (A) Without and (B) with adjustment for adiposity. (TIF) [file pmed.1003017.s002.tif]

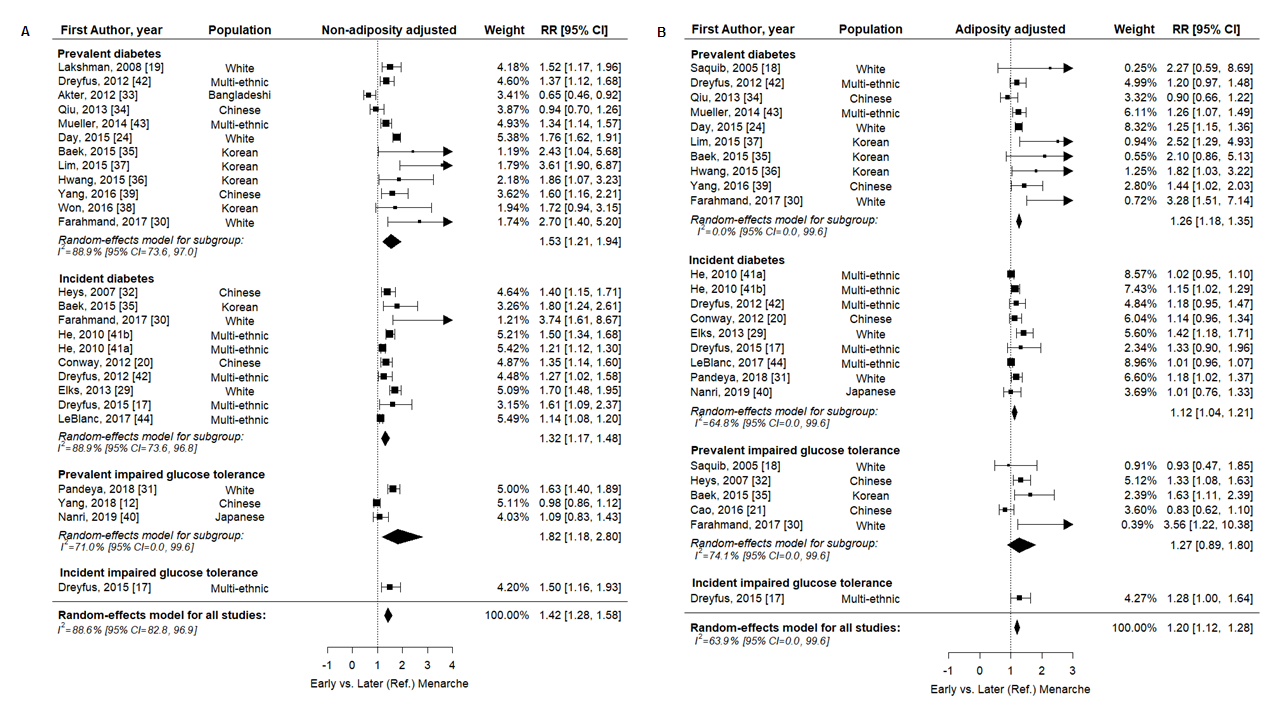

Supplement: S2 Fig — (A) Without and (B) with adjustment for adiposity. (TIF) [file pmed.1003017.s003.tif]

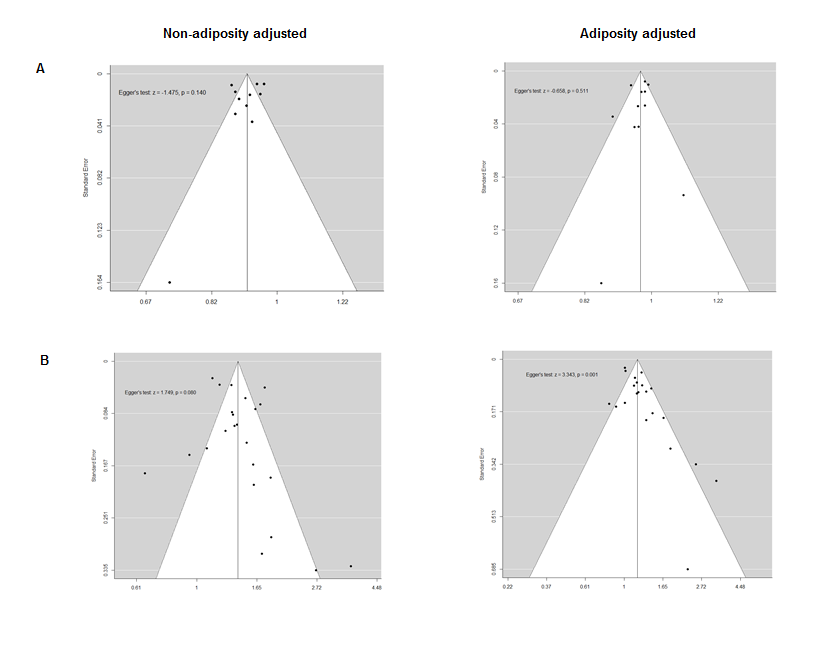

Supplement: S3 Fig — (A) AAM (continuous variable) and (B) early menarche. (TIF) [file pmed.1003017.s004.tif]

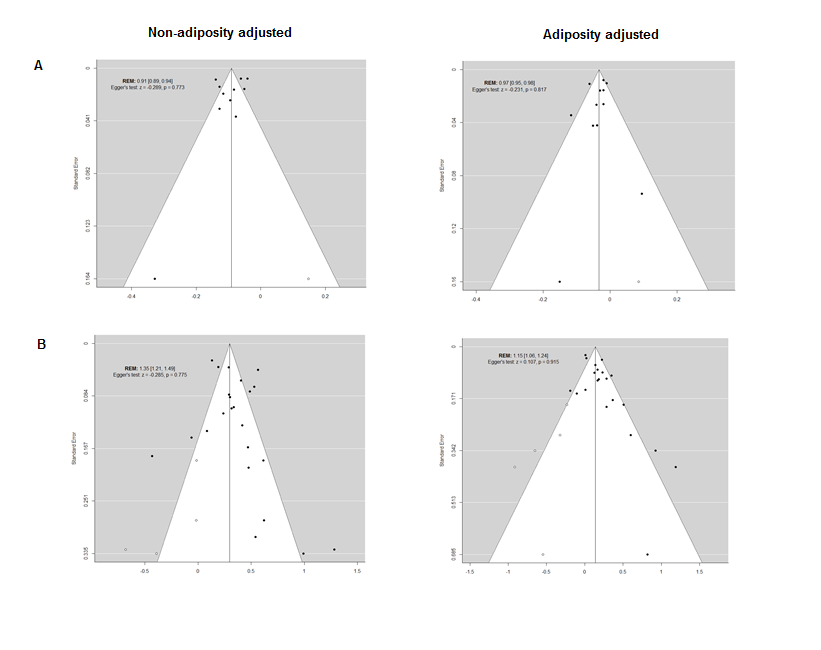

Supplement: S4 Fig — (A) AAM (continuous variable) and (B) early menarche. Open circles indicate filled missing studies. (TIF) [file pmed.1003017.s005.tif]

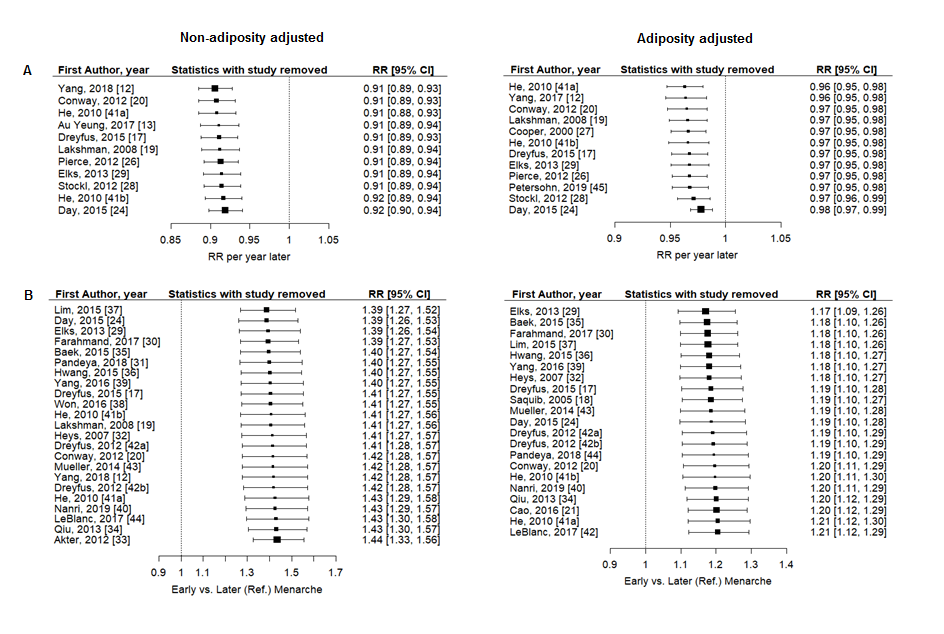

Supplement: S5 Fig — (A) AAM (continuous variable) and (B) early menarche. (TIF) [file pmed.1003017.s006.tif]
