# Supplementary material for: Association of puberty timing with type 2 diabetes: A systematic review and meta-analysis
Source: PLoS Med. 2020 Jan 6;17(1):e1003017. doi: 10.1371/journal.pmed.1003017 (PMC6944335; doi:10.1371/journal.pmed.1003017)
Supplement: S1 Table — (DOCX) [file pmed.1003017.s007.docx]

| **S1 Table. Search strategy in different databases** | | |
| --- | --- | --- |
| Pubmed | Medline | Embase |
| (puberty OR pubertal OR menarche OR Tanner[Text Word] OR (voice (break OR breaking)) OR sexual maturation) AND (diabetes OR diabetic OR insulin OR blood sugar OR glucose OR ((glycated OR glycosylated) AND (haemoglobin OR haemoglobin))) AND (“Epidemiologic studies”[Mesh] OR “case control studies”[Mesh] OR “cohort studies”[Mesh] OR Case control[Text Word] OR cohort stud*[Text Word] OR Cohort analy*[Text Word] OR Follow up stud*[Text Word] OR observational stud*[Text Word] OR (observ*[Text Word] association*[Text Word]) OR Longitudinal[Text Word] OR Retrospective[Text Word] OR Recall*[Text Word] OR Cross sectional[Text Word] OR “Cross-sectional studies”[Mesh] OR (nation*[Text Word] stud*[Text Word]) OR (nation*[Text Word] survey*[Text Word]) OR mendelian randomi*[Text Word]) | 1. puberty.mp. or exp puberty/  2. pubertal.mp.  3. menarche.mp. or exp menarche/  4. Tanner.tw.  5. (voice adj (break or breaking)).mp.  6. sexual maturation.mp. or exp sexual maturation/  7. 1 or 2 or 3 or 4 or 6  8. diabetes.mp. or exp diabetes mellitus/  9. diabetic.mp.  10. exp insulin sensitivity/ or insulin.mp. or exp insulin resistance/  11. blood sugar.mp. or exp glucose blood level/  12. glucose.mp.  13. exp glycosylated hemoglobin/  14. or/8-13  15. 7 and 14  16. Epidemiologic studies/  17. exp case control studies/  18. exp cohort studies/  19. Case control.tw.  20. (cohort adj (study or studies)).tw.  21. Cohort analy$.tw.  22. (Follow up adj (study or studies)).tw.  23. (observational adj (study or studies)).tw.  24. Longitudinal.tw.  25. Retrospective.tw.  26. Cross sectional.tw.  27. Cross-sectional studies/  28. (observ* adj (association or associations)).tw.  29. (nation$ adj (study or studies or survey or surveys)).tw.  30. recall*.tw.  31. exp Mendelian Randomization Analysis/ or mendelian randomi*.mp.  32. or/16-31  33. 15 and 32 | 1. puberty.mp. or exp puberty/  2. pubertal.mp.  3. menarche.mp. or exp menarche/  4. Tanner.tw.  5. (voice adj (break or breaking)).mp.  6. sexual maturation.mp. or exp sexual maturation/  7. 1 or 2 or 3 or 4 or 6  8. diabetes.mp. or exp diabetes mellitus/  9. diabetic.mp.  10. exp insulin sensitivity/ or insulin.mp. or exp insulin resistance/  11. blood sugar.mp. or exp glucose blood level/  12. glucose.mp.  13. exp glycosylated hemoglobin/  14. 8 or 9 or 10 or 11 or 12 or 13  15. 7 and 14  16. clinical study/  17. Case control study/  18. Family study/  19. Longitudinal study/  20. Retrospective study/  21. Prospective study/  22. Cohort analysis/  23. (Cohort adj (study or studies)).mp.  24. (Case control adj (study or studies)).tw.  25. (follow up adj (study or studies)).tw.  26. (observational adj (study or studies)).tw.  27. (epidemiologic$ adj (study or studies)).tw.  28. (cross sectional adj (study or studies)).tw.  29. (observ* adj (association or associations)).tw.  30. (nation$ adj (study or studies or survey or surveys)).tw.  31. recall*.tw.  32. exp Mendelian randomization analysis/ or mendelian randomi*.mp. or exp Mendelian randomization/  33. or/16-32  34. 15 and 33 |
