# Supplementary material for: Association of puberty timing with type 2 diabetes: A systematic review and meta-analysis
Source: PLoS Med. 2020 Jan 6;17(1):e1003017. doi: 10.1371/journal.pmed.1003017 (PMC6944335; doi:10.1371/journal.pmed.1003017)
Supplement: S2 Table — (DOCX) [file pmed.1003017.s008.docx]

| **S2 Table. Summary of eligible studies for prevalent diabetes and/or impaired glucose tolerance** | | | | | | |
| --- | --- | --- | --- | --- | --- | --- |
| First author,  year | Year at  enrolment | Outcome, definition | Controlled variables without anthropometric adiposity measures | | Controlled variables with anthropometric adiposity measures | |
|  |  |  | RR (95% CI) | Adjustment | RR (95% CI) | Adjustment |
| Cooper,  2000 [27] | 1934-1939 | Diabetes: Self-reported physician diagnosis |  |  | 1.1 (0.9, 1.3) per year later | Age and BMI at age 30 |
| Saquib,  2005 [18] | 1984-1987 | Diabetes: fasting glucose≥7.0 mmol/L (≥126 mg/dL), 2-hour glucose≥11.1 mmol/L (≥200 mg/dl), a previous physician diagnosis or use of anti-diabetic medication |  |  | <12 vs. ≥16 (Ref): 2.27 (0.62, 9.09)^a^ | Age, number of pregnancies, exercise≥3 times/week, cigarette smoking, estrogen use, family history of diabetes and BMI |
|  |  | Impaired glucose tolerance: fasting glucose=6.1 mmol/L (110 mg/dL) - 7.0 mmol/L or 2-hour glucose=7.8 mmol/L (140 mg/dL) - 11.1 mmol/L |  |  | <12 vs. ≥16 (Ref): : 0.93 (0.47, 1.85)^a^ | Age, number of pregnancies, exercise≥3 times/week, cigarette smoking, estrogen use, family history of diabetes and BMI |
| Heys,  2007 [32] | 2003-2004 | Impaired glucose tolerance: fasting glucose>5.6 mmol/L or use of anti-diabetic medication | <12.5 vs. ≥14.5 (Ref): 1.40 (1.15, 1.71) | Age, education and number of pregnancies | <12.5 vs. ≥14.5 (Ref): 1.33 (1.08, 1.63) | Previous model + waist circumference |
| Lakshman,  2008 [19] | 1993-1997 | Diabetes: Self-reported physician diagnosis or use of diabetes-specific medication | 0.91 (0.87, 0.96) per year later | Age at baseline, smoking, occupational social class, educational level, physical activity, family history of diabetes, reproductive factors parity, oral contraceptive and use hormone replacement therapy | 0.98 (0.93, 1.03) per year later | Previous model + BMI |
|  |  |  | 8-11 vs. 15-18 (Ref): 1.52 (1.18, 1.96)^a^ | Age at baseline, smoking, occupational social class, educational level, physical activity and family history of diabetes |  |  |
| Akter,  2012 [33] | 2009-2010 | Diabetes: physician diagnosis or anti-diabetic medication and impaired glucose tolerance: fasting glucose≥6.1 mmol/L (≥110 mg/dL) | <12 vs. >13-16 (Ref)  : 0.65 (0.46, 0.93) | Age, education, marital status, use of tobacco products, ever use of contraceptives, and number of pregnancies |  |  |
| Dreyfus,  2012 [42] | 1987-1989 | Diabetes: fasting glucose≥7.0 mmol/L (126 mg/dL), non-fasting glucose>11.1 mmol/L (200 mg/dl), self-reported physician-diagnosis or use of hypoglycemic medication at 30 years or older | 8-11 vs. 13 (Ref)  : 1.37 (1.12, 1.68) | Age at baseline, race, center, family history of diabetes, smoking status, use of oral contraceptives and education | 8-11 vs. 13 (Ref): 1.20 (0.97, 1.48) | Previous model + baseline BMI, height and waist circumference |
| Pierce,  2012 [26] | 1946 | Diabetes: Ever treated with diet or oral hypoglycemic agents, or who had insulin added more than 2 years after diagnosis at 30 years or older | 0.72 (0.52, 0.99) per year later | No | 0.86 (0.63, 1.18) per year later | Adult BMI |
| Stockl,  2012 [28] | 2006-2008 | Diabetes: use of glucose-lowering medication, self-reported physician diagnosis, fasting glucose≥7.0 mmol/L (≥126 mg/dL) or 2-hour glucose≥11.1 mmol/L (≥200 mg/dL) | 0.83 (0.73, 0.95) per year later | Year of birth, physical activity, education, marital status, smoking, alcohol consumption and menopausal status | 0.84 (0.73, 0.98) per year later | Previous model + current BMI |
|  |  | Prediabetes: fasting glucose=6.1 mmol/L - 6.9mmol/L or 2-hour glucose=7.8 mmol/L (140 mg/dL) - 11.1 mmol/L | 0.91 (0.85, 0.98) per year later |  | 0.92 (0.85, 0.99) per year later |  |
|  |  | Diabetes and prediabetes | 0.88 (0.83, 0.94) per year later |  | 0.89 (0.83, 0.95) per year later |  |
| ^a^Results were computed with the reciprocal of risk estimates at highest category  BMI, Body mass index; OR, odds ratio; CI, confidence interval | | | | | | |

| **S2 Table. Summary of eligible studies for prevalent diabetes and/or impaired glucose tolerance (continued)** | | | | | | |
| --- | --- | --- | --- | --- | --- | --- |
| First author,  year | Year at  enrolment | Outcome, definition | Controlled variables without anthropometric adiposity measures | | Controlled variables with anthropometric adiposity measures | |
|  |  |  | RR (95% CI) | Adjustment | RR (95% CI) | Adjustment |
| Qiu,  2013 [34] | 2011-2012 | Diabetes: fasting glucose≥7.0 mmol/L (≥126 mg/dL) or 2-hour glucose≥11.1 mmol/L (≥200 mg/dL), a previous physician diagnosis or use of anti-diabetic medication | 9-14 vs. 16 (Ref): 0.94 (0.70, 1.26) | Age at enrollment, physical activity, parity, smoking, alcohol consumption, family history of diabetes, age at menopause and type of menopause | 9-14 vs. 16 (Ref): 0.90 (0.66, 1.21) | Previous model + BMI and waist circumference |
| Mueller,  2014 [43] | 2008-2010 | Diabetes: self-report diagnosis, use of medication for diabetes, fasting glucose≥126 mg/dL, 2-hour glucose≥200 mg/dL or HbA1c≥6.5% | <11 vs. 13-14 (Ref): 1.34 (1.14, 1.57) | Age at enrollment, study center, race, maternal education, maternal diabetes, paternal diabetes and birth weight | <11 vs. 13-14 (Ref): 1.26 (1.07, 1.49) | Previous model + BMI at age 20 years |
| Baek,  2015 [35] | 2012-2013 | Diabetes: self-report physician diagnosis, use of insulin or hypoglycemic medication, fasting glucose≥126 mg/dL (7.0 mmol/L) or HbA1c≥6.5% (48 mmol/mol) | <13 vs. 13-16 (Ref): 2.43 (1.04, 5.69) | Age at enrollment, education, spouse, income, parity, menopause status, smoking, alcohol and physical activity | <13 vs. 13-16 (Ref): 2.10 (0.86, 5.14) | Previous model + BMI |
|  |  | Prediabetes: fasting glucose=100-125 mg/dL (5.6mmol/L-6.9 mmol/L) or HbA1c=5.7%-6.4% (39-46 mmol/mol) | <13 vs. 13-16 (Ref): 1.80 (1.24, 2.61) |  | <13 vs. 13-16 (Ref): 1.63 (1.11, 2.39) |  |
|  |  | Diabetes + Prediabetes | 13-16 (Ref),  <13: 1.85 (1.28, 2.66) |  | 13-16 (Ref),  <13: 1.66 (1.14, 2.41) |  |
| Day,  2015 [24] | 2006-2010 | Diabetes: Self-report physician diagnosis, excluding possible type 1 diabetes (based on age at diagnosis≤35, use of insulin within 1 year of diagnosis or diagnosis less than 1 year before enrollment) | 0.87 (0.85, 0.88) per year later;  8-11 vs. 13-14 (Ref): 1.76 (1.62, 1.91) | Birth year, age and age-squared | 0.94 (0.92, 0.96) per year later; 8-11 vs. 13-14 (Ref): 1.25 (1.15, 1.36) | Previous model + socioeconomic position (11 principle components) and adiposity/body composition (5 principle components) |
|  |  |  | relatively younger vs. about average (Ref): 1.44 (1.30, 1.59) |  | relatively younger vs. about average (Ref): 1.24 (1.11, 1.37) |  |
| Hwang,  2015 [36] | 2007-2009 | Diabetes: Self-report physician diagnosis | 10-12 vs. 13-15 (Ref): 1.86 (1.07, 3.23) | Age, education, income, use of hormonal medication, smoking status, alcohol use, exercise status and diagnosis of hypertension, dyslipidemia and cardiac disease | 10-12 vs. 13-15 (Ref): 1.82 (1.03, 3.23) | Previous model + BMI and waist circumference |
| Lim,  2015 [37] | 2007-2009 | Diabetes: use of a glucose-lowering medication, self-report physician diagnosis, fasting glucose≥126 mg/dL | <12 vs. ≥12 (Ref): 3.61 (1.90, 6.88) | Age | <12 vs. ≥12 (Ref): 2.52 (1.29, 4.94) | Previous model + BMI |
| Cao,  2016 [21] | 2011-2014 | Impaired glucose tolerance: fasting glucose≥5.6 mmol/L |  |  | 11-13vs. 16-20 (Ref): 0.83 (0.62, 1.10)^a^ | Age, education level, physical activity and BMI |
| Won,  2016 [38] | 2010-2013 | Diabetes: Self-reported physician diagnosis | <11 vs. ≥17 (Ref): 1.72 (0.94, 3.15) | Age, current smoking, college graduation and menstruation |  |  |
| ^a^Results were computed with the reciprocal of risk estimates at highest category  BMI, Body mass index; OR, odds ratio; CI, confidence interval | | | | | | |

| **S2 Table. Summary of eligible studies for prevalent diabetes and/or impaired glucose tolerance (continued)** | | | | | | |
| --- | --- | --- | --- | --- | --- | --- |
| First author,  year | Year at  enrolment | Outcome, definition | Controlled variables without anthropometric adiposity measures | | Controlled variables with anthropometric adiposity measures | |
|  |  |  | RR (95% CI) | Adjustment | RR (95% CI) | Adjustment |
| Yang,  2016 [39] | 2011-2013 | Diabetes: use of anti-diabetic medication, fasting glucose ≥7.0 mmol/L (126 mg/dl.) | ≤12 vs. 15-16 (Ref): 1.60 (1.16, 2.22) | Age | ≤12 vs. 15-16 (Ref): 1.44 (1.02, 2.03) | Age, education, marital status, occupation, smoking status, drinking, hypertension, abnormal lipid, family history of diabetes, age at menopause and BMI |
| Au Yeung, 2017 [13] | 2003-2008 | Diabetes: use of anti-diabetic medication, fasting glucose ≥7.0 mmol/L (126 mg/dl.) | 0.65 (0.32, 1.33) per year later | Mendelian Randomization |  |  |
|  |  |  | 0.92 (0.89, 0.95) per year later | Education, recruitment phase, age, smoking, alcohol use, physical activity, job type, corresponding medications such as antihypertensive for blood pressure |  |  |
| Farahmand, 2017 [30] | 1998 | Diabetes: fasting glucose≥7.0 mmol/L (126mg/dL), 2-hour glucose≥11.1 mmol/L (200 mg/dL) | <11 vs. 13-14 (Ref): 2.70 (1.40, 5.20) | Family history of diabetes, parity, education and age | <11 vs. 13-14 (Ref): 3.28 (1.50, 7.10) | Previous model + BMI |
|  |  | Prediabetes: fasting glucose=100 mg/dL (5.6 mmol/L) -125 mg/dL (6.9 mmol/L) or 2-hour glucose=140 mg/dL (7.8 mmol/L) -199 mg/dL (11.0 mmol/L) | <11 vs. 13-14 (Ref): 3.74 (1.60, 8.60) |  | <11 vs. 13-14 (Ref): 3.56 (1.20, 10.20) |  |
| Petersohn, 2019 [45] | 1999-2000 | Diabetes: self-report physician diagnosis or random blood glucose>200 mg/dL |  |  | 0.95 (0.83, 0.98) per year later | BMI, age BMI-age interaction, family history of diabetes |
| ^a^Results were computed with the reciprocal of risk estimates at highest category  BMI, Body mass index; OR, odds ratio; CI, confidence interval | | | | | | |
