# Supplementary material for: Association of puberty timing with type 2 diabetes: A systematic review and meta-analysis
Source: PLoS Med. 2020 Jan 6;17(1):e1003017. doi: 10.1371/journal.pmed.1003017 (PMC6944335; doi:10.1371/journal.pmed.1003017)
Supplement: S3 Table — (DOCX) [file pmed.1003017.s009.docx]

| **S3 Table. Summary of eligible studies for incident diabetes and/or impaired glucose tolerance** | | | | | | |
| --- | --- | --- | --- | --- | --- | --- |
| First author,  year | Year at  enrolment | Outcome, definition | Controlled variables without anthropometric adiposity measures | | Controlled variables with anthropometric adiposity measures | |
|  |  |  | RR (95% CI) | Adjustment | RR (95% CI) | Adjustment |
| He,  2010 [41] | 1980 | Diabetes: fasting glucose≥7.8 mmol/L (≥140 mg/dL) or ≥7.0 mmol/L (≥126 mg/dL) (after 1997), 2-hour/ random glucose≥ 11.1mmol/L (≥200 mg/dL), at least one symptom related to diabetes (excessive thirst, polyuria, weight loss, hunger) or treatment with insulin/ oral hypoglycemic medication | 0.94 (0.92, 0.95) per year later;  ≤11 vs. 13 (Ref): 1.21 (1.13, 1.31) | Age groups, birth weight, having been breastfed, childhood socioeconomic status, ethnicity, family history of diabetes, perceived body figure at age 10 years, the baseline factors physical activity, quintile of dietary score, alcohol consumption, smoking status, hypertension, hypercholesterolemia, menopause status, use of hormone replacement therapy, adult socioeconomic status, reproductive factors (parity, oral contraceptive use, and regularity of menstrual cycles at ages 18–22 years) | 0.99 (0.97, 1.01) per year later;  13 (Ref),  ≤11 vs. 13 (Ref): 1.02 (0.95, 1.10) | Previous model + BMI over the course of follow-up |
|  | 1991 |  | 0.88 (0.86, 0.91) per year later;  ≤11 vs. 13 (Ref): 1.50 (1.34, 1.69) |  | 0.97 (0.94, 1.00) per year later;  ≤11 vs. 13 (Ref): (1.02, 1.29) |  |
| Conway,  2012 [20] | 1997-2000 | Diabetes: fasting glucose≥7 mmol/L, oral glucose tolerance test≥11.1 mmol/L and/or use of a hypoglycemic agent | 0.95 (0.92, 0.98) per year later;  8-13 vs. 17-26 (Ref): 1.35 (1.14, 1.59)^a^ | Birth cohort, education and income | 0.98 (0.95, 1.01) per year later; 8-13 vs. 17-26 (Ref): 1.14 (0.95, 1.33)^a^ | Previous model + participation in team sports during adolescence, BMI at baseline and BMI at age 20 |
| Dreyfus,  2012 [42] | 1987-1989 |  | 8-13 vs. 17-26 (Ref): 1.27 (1.02, 1.58) |  | 8-13 vs. 17-26 (Ref): 1.18 (0.95, 1.47) |  |
| Elks,  2013 [29] | 1991 | Diabetes: self-report physician diagnosis, confirmed by medical records, or local and national diabetes and pharmaceutical registers | 0.89 (0.86, 0.93) per year later;  8-11 vs. 13 (Ref): 1.70 (1.48, 1.94) | Age at recruitment, date of birth, center, age at first full-term pregnancy, parity, menopausal status, use of oral contraceptive pill, use of hormone replacement therapy | 0.96 (0.91, 1.01) per year later;  8-11 vs. 13 (Ref): 1.42 (1.18, 1.71) | Previous model + adult BMI |
| Dreyfus,  2015 [17] | 1985 | Diabetes: fasting glucose≥7.0 mmol/L (126 mg/dL), HbA1c≥6.5%, 2-hour glucose≥11.1 mmol/L (200 mg/dL), or use of diabetes medication | 0.93 (0.86, 1.00) per year later^b^,  8-11 vs. 14-17 (Ref): 1.61 (1.09, 2.37) | Age, center, race, parental history of diabetes, education, pre-high school physical activity, high school physical activity, smoking status, oral contraceptive use, physical activity and alcohol intake | 0.90 (0.86, 0.94) per year later^b^, 14-17 (Ref),  8-11 vs. 14-17 (Ref): 1.33 (0.90, 1.96) | Previous model + baseline BMI |
|  |  | Impaired glucose tolerance: fasting glucose=5.6 (100 mg/dL) -6.9 mmol/L, not taking diabetes medication | 0.96 (0.89, 1.05) per year later^b^,  8-11 vs. 14-17 (Ref): 1.50 (1.17, 1.94) |  | 0.93 (0.88, 0.98) per year later^b^,  8-11 vs. 14-17 (Ref): 1.28 (0.99, 1.62) |  |
| ^a^Results were computed with the reciprocal of risk estimates at highest category  ^b^Results were computed with the reciprocal of risk estimates per year early age at menarche  BMI, Body mass index; OR, odds ratio; CI, confidence interval | | | | | | |

| **S3 Table. Summary of eligible studies for incident diabetes and/or impaired glucose tolerance (continued)** | | | | | | |
| --- | --- | --- | --- | --- | --- | --- |
| First author,  year | Year at  enrolment | Outcome, definition | Controlled variables without anthropometric adiposity measures | | Controlled variables with anthropometric adiposity measures | |
|  |  |  | RR (95% CI) | Adjustment | RR (95% CI) | Adjustment |
| LeBlanc,  2017 [44] | 1993-1998 | Diabetes: self-report diagnosis, use of diabetes medications | <12 vs. 12 (Ref): 1.14 (1.08, 1.20) | Age | <12 vs. 12 (Ref): 1.01 (0.95, 1.06) | Age, race, hormone therapy intervention arm membership, baseline physical activity, baseline alcohol consumption, baseline smoking history, education, baseline marital status, number of term pregnancies, family history of diabetes, years since menopause at baseline, baseline waist circumference, history of oral contraceptive use at baseline, baseline metformin use and baseline BMI |
| Yang,  2018 [12] | 2004-2008 | Diabetes: Data linkage with the nationwide health insurance system | 0.96 (0.94, 0.97) per year later;  13 vs. ≥18 (Ref): 1.33 (1.24, 1.44)^a^ | Education, household income, smoking status, alcohol intake, blood pressure, physical activity, menopause status, parity, age at first birth, breastfeeding duration per child, and oral contraceptive use | 0.98 (0.97, 1.00) per year later | Previous model + baseline BMI and waist circumference |
| Pandeya,  2018 [31] | 1985-2009 | Diabetes: self-report physician diagnosis or information from hospital patient registry data | ≤10 vs. 13 (Ref): 1.63 (1.40, 1.89) | Women's year of birth, age at baseline, education,  smoking status at baseline, number of children, age at first birth, menopausal status/timing and hormone therapy at baseline | ≤10 vs. 13 (Ref): 1.18 (1.02, 1.37) | Previous model + baseline BMI |
| Nanri,  2019 [40] | 1990, 1993 | Diabetes: self-reported physician diagnosis by examining medical records | ≤13 vs. ≥16 (Ref): 1.09 (0.83, 1.43)^a^ | Age, study area, smoking status , alcohol consumption , family history of diabetes mellitus, total physical activity, history of hypertension, total energy intake, coffee consumption, energy-adjusted daily intake of foods or nutrients | ≤13 vs. ≥16 (Ref): 1.01 (0.76, 1.33)^a^ | Previous model + BMI |
| ^a^Results were computed with the reciprocal of risk estimates at highest category  BMI, Body mass index; OR, odds ratio; CI, confidence interval | | | | | | |
