# Supplementary material for: Association of puberty timing with type 2 diabetes: A systematic review and meta-analysis
Source: PLoS Med. 2020 Jan 6;17(1):e1003017. doi: 10.1371/journal.pmed.1003017 (PMC6944335; doi:10.1371/journal.pmed.1003017)
Supplement: S4 Table — (DOCX) [file pmed.1003017.s010.docx]

| **S4 Table. Quality of eligible studies for prevalent diabetes/ impaired glucose tolerance assessed by The Newcastle-Ottawa Quality Assessment Scale for cohort studies** | | | | | | | | | | | | | | | | | | | | | |
| --- | --- | --- | --- | --- | --- | --- | --- | --- | --- | --- | --- | --- | --- | --- | --- | --- | --- | --- | --- | --- | --- |
| First author, year | Cooper,  2000  [27] | Pierce,  2012  [26] | Saquib, 2015 [18] | Heys,  2007  [32] | Lakshman,  2008  [19] | Akter,  2012  [33] | Dreyfus,  2012  [42] | Stockl,  2012  [28] | Qiu,  2012  [34] | Mueller,  2014  [43] | Baek,  2015  [35] | Day,  2015  [24] | Hwang,  2015  [36] | Lim,  2015  [37] | Cao,  2016  [21] | Won,  2016  [38] | Yang,  2016  [39] | Au Yeung,  2017  [13] | Farahmand,  2017  [30] | Petersohn,  2019  [45] |  |
| 1. Truly or somewhat representative of the general population | 0 | 1 | 1 | 1 | 1 | 1 | 1 | 1 | 1 | 1 | 1 | 1 | 1 | 1 | 1 | 1 | 0 | 1 | 1 | 1 |  |
| 1. Selection of the non-exposed cohort from the same community as the exposed cohort | 1 | 1 | 1 | 1 | 1 | 1 | 1 | 1 | 1 | 1 | 1 | 1 | 1 | 1 | 1 | 1 | 1 | 1 | 1 | 1 |  |
| 1. At least some description of assessment | 1 | 1 | 1 | 1 | 1 | 1 | 1 | 1 | 1 | 1 | 1 | 1 | 1 | 1 | 0 | 1 | 1 | 1 | 1 | 1 |  |
| 1. Demonstration that the outcome was not present at the start of study | 1 | 1 | NA | NA | NA | NA | NA | NA | NA | NA | NA | NA | NA | NA | NA | NA | NA | NA | NA | NA |  |
| 5a) Controls for age | 1 | 0 | 1 | 1 | 1 | 1 | 1 | 1 | 1 | 1 | 1 | 1 | 1 | 1 | 1 | 1 | 1 | 1 | 1 | 1 |  |
| 5b) Controls for additional factors (ethnicity, diet, physical activity) | 0 | 0 | 1 | 0 | 1 | 0 | 0 | 1 | 1 | 0 | 1 | 0 | 1 | 0 | 1 | 0 | 0 | 1 | 0 | 0 |  |
| 1. Assessment of outcome – oral glucose tolerance test or record linkage | 0 | 0 | 1 | 1 | 0 | 1 | 1 | 1 | 1 | 1 | 1 | 0 | 0 | 1 | 1 | 0 | 1 | 1 | 1 | 1 |  |
| 1. At least 5 years follow-up for outcomes to occur | 1 | 1 | NA | NA | NA | NA | NA | NA | NA | NA | NA | NA | NA | NA | NA | NA | NA | NA | NA | NA |  |
| 1. Adequate ≥70% of original cohort | 0 | 0 | 0 | 1 | 0 | 1 | 1 | 0 | 1 | 1 | 1 | 1 | 1 | 1 | 1 | 1 | 1 | 1 | 1 | 1 |  |
| Total (max 7 or 9) | 5 | 5 | 6 | 6 | 5 | 6 | 6 | 6 | 7 | 6 | 7 | 5 | 6 | 6 | 6 | 5 | 5 | 7 | 6 | 6 |  |
