# Supplementary material for: Association of puberty timing with type 2 diabetes: A systematic review and meta-analysis
Source: PLoS Med. 2020 Jan 6;17(1):e1003017. doi: 10.1371/journal.pmed.1003017 (PMC6944335; doi:10.1371/journal.pmed.1003017)
Supplement: S5 Table — (DOCX) [file pmed.1003017.s011.docx]

| **S5 Table. Quality of eligible studies for incident diabetes/ impaired glucose tolerance assessed by The Newcastle-Ottawa Quality Assessment Scale for cohort studies** | | | | | | | | | |
| --- | --- | --- | --- | --- | --- | --- | --- | --- | --- |
| First author, year | He  2010  [41] | Conway,  2012  [20] | Dreyfus,  2012  [42] | Elks,  2013  [29] | Dreyfus,  2015  [17] | LeBlanc,  2017  [44] | Yang,  2018  [12] | Pandeya,  2018  [31] | Nanri,  2019  [40] |
| 1. Truly or somewhat representative of the general population | 1 | 1 | 1 | 1 | 1 | 1 | 1 | 1 | 1 |
| 1. Selection of the non-exposed cohort from the same community as the exposed cohort | 1 | 1 | 1 | 1 | 1 | 1 | 1 | 1 | 1 |
| 1. At least some description of assessment | 1 | 1 | 1 | 1 | 1 | 1 | 1 | 1 | 1 |
| 1. Demonstration that the outcome was not present at the start of study | 1 | 1 | 1 | 1 | 1 | 1 | 1 | 1 | 1 |
| 5a) Controls for age | 1 | 1 | 1 | 1 | 1 | 1 | 1 | 1 | 1 |
| 5b) Controls for additional factors (ethnicity, diet, physical activity) | 1 | 1 | 0 | 0 | 1 | 1 | 1 | 0 | 1 |
| 6) Assessment of outcome – oral glucose tolerance test or record linkage | 1 | 1 | 1 | 1 | 1 | 0 | 1 | 1 | 1 |
| 1. At least 5 years follow-up for outcomes to occur | 1 | 1 | 1 | 1 | 1 | 1 | 1 | 1 | 1 |
| 1. Adequate ≥70% of original cohort | 1 | 1 | 1 | 1 | 1 | 1 | 1 | 1 | 0 |
| Total (max 9) | 9 | 9 | 8 | 8 | 9 | 8 | 9 | 8 | 8 |
