# Supplementary material for: Association of puberty timing with type 2 diabetes: A systematic review and meta-analysis
Source: PLoS Med. 2020 Jan 6;17(1):e1003017. doi: 10.1371/journal.pmed.1003017 (PMC6944335; doi:10.1371/journal.pmed.1003017)
Supplement: S6 Table — (DOCX) [file pmed.1003017.s012.docx]

| **S6 Table. Univariable meta-regression results and pooled RR for diabetes and glucose intolerance in study subgroups** | | | | | | | | | | | | | | | | | | | | |
| --- | --- | --- | --- | --- | --- | --- | --- | --- | --- | --- | --- | --- | --- | --- | --- | --- | --- | --- | --- | --- |
| Factors | RR per year later age at menarche | | | | | | | | |  | Early versus later (Ref.) menarche | | | | | | | | | |
|  | Non-adiposity adjusted | | | |  | Adiposity adjusted | | | |  | Non-adiposity adjusted | | | |  | Adiposity adjusted | | | | |
|  | N | RR (95% CI) | P value^a^ | R^2^ (%) |  | N | RR (95% CI) | P value^a^ | R^2^ (%) |  | N | RR (95% CI) | P value^a^ | R^2^ (%) |  | N | RR (95% CI) | P value^a^ | R^2^ (%) |  |
| Year of enrolment |  |  |  | 0 |  |  |  |  | 1.3 |  |  |  |  | 0 |  |  |  |  | 11.8 |  |
| until 2000 | 7 | 0.91 (0.89, 0.94) | - |  |  | 9 | 0.98 (0.97, 0.99) | - |  |  | 12 | 1.39 (1.26, 1.53) | - |  |  | 12 | 1.14 (1.06, 1.23) | - |  |  |
| after 2000 | 4 | 0.91 (0.87, 0.95) | 0.868 |  |  | 3 | 0.94 (0.90, 0.99) | 0.139 |  |  | 11 | 1.40 (1.11, 1.78) | 0.809 |  |  | 9 | 1.28 (1.08, 1.51) | 0.266 |  |  |
|  |  |  |  |  |  |  |  |  |  |  |  |  |  |  |  |  |  |  |  |  |
| Age at outcome assessment, years |  |  |  | 3.1 |  |  |  |  | 0 |  |  |  |  | 0 |  |  |  |  | 0 |  |
| <50 | 1 | 0.88 (0.86, 0.91) | - |  |  | 2 | 0.97 (0.94, 1.00) | - |  |  | 7 | 1.66 (1.13, 2.45) | - |  |  | 5 | 1.65 (1.18. 2.29) | - |  |  |
| ≥50 | 10 | 0.92 (0.89, 0.94) | 0.297 |  |  | 10 | 0.97 (0.95, 0.99) | 0.939 |  |  | 16 | 1.34 (1.26, 1.48) | 0.112 |  |  | 16 | 1.15 (1.07, 1.24) | 0.034 |  |  |
|  |  |  |  |  |  |  |  |  |  |  |  |  |  |  |  |  |  |  |  |  |
| Number of variables adjusted |  |  |  | 0 |  |  |  |  | 0 |  |  |  |  | 0 |  |  |  |  | 0 |  |
| <5 | 3 | 0.90 (0.82, 0.98) | - |  |  | 3 | 0.98 (0.88, 1.08) | - |  |  | 8 | 1.59 (1.30, 1.95) | - |  |  | 3 | 1.32 (0.74, 2.35) | - |  |  |
| ≥5 | 8 | 0.92 (0.89, 0.94) | 0.617 |  |  | 9 | 0.97 (0.95, 0.98) | 0.963 |  |  | 15 | 1.31 (1.15, 1.49) | 0.248 |  |  | 18 | 1.18 (1.10, 1.27) | 0.973 |  |  |
|  |  |  |  |  |  |  |  |  |  |  |  |  |  |  |  |  |  |  |  |  |
| Early menarche, years |  |  |  |  |  |  |  |  |  |  |  |  |  | 27.8 |  |  |  |  | 0 |  |
| <12 |  | - |  |  |  |  | - |  |  |  | 12 | 1.50 (1.37, 1.64) | - |  |  | 10 | 1.20 (1.11, 1.30) | - |  |  |
| <14 |  | - |  |  |  |  | - |  |  |  | 11 | 1.29 (1.03, 1.60) | 0.045 |  |  | 11 | 1.20 (1.02, 1.41) | 0.482 |  |  |
|  |  |  |  |  |  |  |  |  |  |  |  |  |  |  |  |  |  |  |  |  |
| Reference AAM category, years |  |  |  |  |  |  |  |  |  |  |  |  |  | 0 |  |  |  |  | 0 |  |
| ≥12 |  | - |  |  |  |  | - |  |  |  | 15 | 1.43 (1.22, 1.67) | - |  |  | 13 | 1.22 (1.12, 1.34) | - |  |  |
| ≥14 |  | - |  |  |  |  | - |  |  |  | 8 | 1.34 (1.18, 1.52) | 0.702 |  |  | 8 | 1.12 (0.97, 1.30) | 0.297 |  |  |
|  |  |  |  |  |  |  |  |  |  |  |  |  |  |  |  |  |  |  |  |  |
| Measures of association |  |  |  | 12.2 |  |  |  |  | 13.2 |  |  |  |  | 0 |  |  |  |  | 0 |  |
| Hazards ratio | 5 | 0.93 (0.90, 0.97) | - |  |  | 5 | 0.98 (0.97, 0.99) | - |  |  | 5 | 1.30 (1.06, 1.60) | - |  |  | 4 | 1.18 (0.99, 1.40) | - |  |  |
| Odds ratio | 5 | 0.90 (0.88, 0.93) | 0.212 |  |  | 5 | 0.97 (0.95, 1.00) | 0.762 |  |  | 16 | 1.43 (1.22, 1.67) | 0.538 |  |  | 15 | 1.21 (1.08, 1.35) | 0.817 |  |  |
| Relative risks | 1 | 0.88 (0.83, 0.94) | 0.246 |  |  | 2 | 0.92 (0.86, 0.98) | 0.044 |  |  | 2 | 1.48 (1.22, 1.79) | 0.559 |  |  | 2 | 1.21 (1.09, 1.36) | 0.790 |  |  |
| ^a^The reference category in meta-regression is the first subgroup in each factor  N, number of estimates, R^2^ (%), % heterogeneity explained | | | | | | | | | | | | | | | | | | | | |
